# Creating a low-cost virtual reality surgical simulation to increase surgical oncology capacity and capability

**DOI:** 10.3332/ecancer.2019.910

**Published:** 2019-03-18

**Authors:** Groesbeck Parham, Eric G Bing, Anthony Cuevas, Boris Fisher, Jonathan Skinner, Mulindi Mwanahamuntu, Richard Sullivan

**Affiliations:** 1 Department of Obstetrics and Gynecology, UNC School of Medicine, University of North Carolina at Chapel, Chapel Hill, NC, USA; 2 Department of Obstetrics and Gynecology, University of Zambia, UTH-Women and Newborn Hospital, Lusaka 10101, Zambia; 3 Center for Global Health Impact, Department of Applied Physiology and Wellness, Simmons School of Education and Human Development, Dallas, TX 75205 USA; 4 Department of Anthropology, Dedman College of Humanities and Sciences, Southern Methodist University, Dallas, TX 75205 USA; 5 Simulation Laboratory, Department of Teaching and Learning, Simmons School of Education and Human Development, Southern Methodist University, TX 75205 USA; 6 Guildhall, Southern Methodist University, TX 75205 USA; 7 Kings Health Partners Comprehensive Cancer Centre, School of Cancer Sciences, Institute of Cancer Policy, King’s College London, London SE1 9RT, UK; * Groesbeck Parham and Eric G Bing are the joint first authors.

**Keywords:** Zambia, cervical cancer, virtual reality, surgical simulation, capacity building, pelvic surgery

## Abstract

Worldwide, more than 80% of people with cancer will require surgery during their disease course, but less than 25% have access to safe, affordable and timely surgery. Among the barriers to increasing surgical capacity are the time and costs required to train novices. Virtual reality (VR) surgical simulations can reduce the time required for novices to reach surgical proficiency, though their costs may exceed USD $100,000. The goal of this study was to determine if a low-cost system, using commercially available technology designed for in-home computer gaming, could be used to create a realistic VR surgical oncology simulation. Standard commercially available VR software and Oculus Rift hardware have been used to provide high-quality visuals and believable surgeon hand interactions. Near identical VR reproduction of an operating room using 1:1 scale matching of real-world elements, including equipment, instruments, supplies and sounds, maintaining frame rate greater than 60 fps to maintain visual fidelity has been created. Internal anatomy was designed as VR replica of human female pelvic anatomy, including organs, veins and other vessels, peritoneum and connective tissue. Internal anatomy was designed to run at 120 fps and to allow for a realistic abdominal radical hysterectomy simulation. Surgical hands were modelled to scale for those with large and small hands. Multiple hand positions were simulated using Oculus touch hardware. Reconstructing the virtual environment to simulate reality as accurately as possible was done to immerse users in the simulator so that they focus on learning and practise without distractions. Training modules were co-designed by experts in learning sciences, human behaviour, VR and gynaecologic oncology. We have successfully created a low-cost VR simulation to help prepare novice surgeons to perform a radical abdominal hysterectomy surgery procedure. The simulation can be used with commercially available computer gaming hardware that currently costs less than USD $1,500. Low-cost VR simulation has the potential to reduce the time and cost to train surgeons to perform surgical oncology procedures, as well as both improve and audit quality. If effective in real-world clinical trials, such simulations have relevance to multiple surgical procedures and applicability in both resource-limited and high-income settings.

## Augmented and virtual reality for surgery

Immersive, highly visual and 3-D virtual reality (VR) simulators are designed to closely replicate real life. VR simulators are technically advanced and are available in a range of sizes and shapes. Some incorporate gaming-industry headsets and cellular phones, while others involve large-scale simulators resembling carnival rides. The field of VR training in healthcare is growing exponentially. The Virtual Reality Modelling Language was first introduced in 1994 and was intended for the development of ‘virtual worlds’ without dependency on headsets. All modern VR displays are based on technology developed for smartphones, including gyroscopes and motion sensors for tracking head, hand and body positions; small HD screens for stereoscopic displays; and small, lightweight and fast processors. These components led to relative affordability for independent VR developers and led in 2012 to Oculus Rift offering the first independently developed VR headset. Over the last decade, both the military and the games industry have driven the development of increasingly immersive VR and its application has been astonishingly wide from flight simulation and astronaut training through to mechanical engineering and architectural design. Some VR is created for stand-alone computers, whilst others run as networked VR ‘cyberspace’. VR shares some elements with ‘augmented reality’ (AR) that blends what the user sees in their real surroundings with digital content generated by computer software.

To date, AR has been the main focus for surgery due to a combination of advances in medical imaging and the increasing availability of intraoperative sensors. High-resolution endoscopes, real-time tracking of instruments, as well as instrument recognition via radio-frequency identification (RFID) and image processing generate large amounts of information. AR has emerged as the main interface paradigm. This approach has become increasingly sophisticated, overcoming many of the earlier obstacles where AR, even with high accuracy registration and advanced visualisation metaphors still overloaded surgeons. Context-aware visualisations have now gained greater traction with the advent of minimally invasive surgical techniques to provide context-aware systems that build easily into the workflow [1]. Today, a range of sophisticated AR approaches are being utilised, including see-through projection systems with smart glasses or a headset with or without tracking; projection systems using a half-silvered mirror; systems to project images directly onto the patient and systems to project digital data onto a display monitor. Technical developments have been driven by the standardisation of 3-D digital formats and the availability of open-source volume-rendering software, as well as now visualisation tools, digital acquisition (4K cameras) and more recently, 3-D printing (3-D printers), leading to greater pan-disease utility and potential application across a range of resource-rich and limited environment’s [2]. However, the major drawbacks have been centred around the lack of clinical testing to demonstrate genuine clinical utility with a recent review finding only 13 relevant clinical studies over the last decade [3].

Whilst AR has been developed to assist with surgery, the focus on VR has been to create an immersive experience that mimics, with high fidelity, the environment and required processes. Orthopaedics and urology have been early adopters of VR. The former has been driven by the need for greater volume experience through the creation of modern arthroscopic simulator training models. However, the clinical evidence for VR clinical utility remains mixed [4] in the most part because of the lack of rigorous educational research, particularly the development and testing of ‘task lists’ to incorporate surgical VR and establish efficacy [5]. Indeed, the use of VR as a training tool for minimally invasive surgery (MIS) was considered over 10 years ago as an important adjunct to surgical training, particularly using high-fidelity simulators, and with the evolution of technology over time, low-cost systems were predicated to provide even greater impact on the surgical landscape [6]. Beyond MIS, the rise of robotics, particularly in urology (Da Vinci systems), has been a major driver for more expensive VR using haptic feedback to mimic the robotic systems [7]. In particular, it has been found that VR appears to improve the learning curve of naïve surgeons performing vesicourethral anastomosis during robot-assisted radical prostatectomy [8].

Reflecting on a decade of AR/VR development in surgery, the trend has been a focus on high-income needs and affluent settings (MIS and robotics) at the expense of more affordable applications, particularly for VR. Even AR development has followed a more networked cyberspace model that requires good bandwidth connectivity and fiscal resources to pay for the Gb ingress and egress. Furthermore, VR has tended to follow the trajectory of surgical training in high-income countries with its relative paucity of open procedures. VR has not been developed for open procedures which still make up the majority of approaches for cancer surgery in resource-constrained settings. Two major commissions in surgery and cancer surgery noted that the lacunae in global surgery were disproportionality being faced by the most resource-challenged countries [9, 10]. The deficit in surgeons (both general and pelvic/gynaecological), as well as anaesthetists required radical solutions to both capacity and capability that could be affordably scaled-up. This requires the application of VR surgical simulation (VRSS) to open procedures. Less than 5% of lower-income countries and only 20% of middle-income countries have access to basic cancer surgery, mainly because of the lack of skilled workforce. These Commissions specifically noted the need for a focus on selective general, pelvic and oncological capacity building through novel training approaches. Here we describe a new approach to high-fidelity VRSS training for a mainstream, but complex, oncological procedure—radical abdominal hysterectomy (RAH)—for cancer of the cervix, a major surgical need in low- and middle-income settings.

## Virtual reality-enhanced training for cancer in emerging and low-income economies

Cancer, the world’s leading cause of death, is projected to increase at a staggering rate. Most of the increase will occur in the world’s poorest regions. Africa will be hardest hit, and it is estimated that by 2030, cancer will kill over 1 million Africans each year. Within Africa, women will bear the heaviest burden because cervical and breast cancers are its most common malignancies and causes of cancer-related death. One in every five of the world’s annual cervical cancer deaths takes place in sub-Saharan Africa [11], making it the global killing field of cervical cancer [12]. In response to Zambia’s excessive cervical cancer burden, a novel service delivery platform integrated with the country’s public-sector health system—the Cervical Cancer Prevention Programme in Zambia (CCPPZ)—was established [13, 14]. The programme has been scaled-up across the country over the past decade, with over 600,000 women screened in 10 provinces [15]. Approximately 1.2% of women screened through CCPPZ clinics have invasive cervical cancer detected at screening, > 50% of which are early stage and highly curable with surgery. However, the surgical oncology workforce in Zambia, like most sub-Saharan countries in continental Africa, is vastly inadequate to provide care for these women. As a result, women in desperate need of surgery typically spend 4–6 months on a surgical waiting list, during which time their tumours may progress, necessitating more extensive treatment, and ultimately leading to poorer survival [16]. As more countries in Africa and other lower- and middle-income (LMI) settings implement and scale-up cervical cancer screening programmes, they will encounter similar, if not more severe treatment bottlenecks, particularly in surgery and radiotherapy.

There is emerging evidence from research conducted in more developed countries that VR simulation and web-based telemonitoring can accelerate surgical skills transfer. The simulator aims to create ‘pre-trained novices’ by helping them acquire the psycho-motor skills, sensory acuity and to a lesser extent, cognitive planning required to achieve the surgical dexterity necessary to perform complex surgical tasks [6]. Several studies have shown that simulator-based training reduced the time to develop surgical proficiency in the operating room (OR) [17, 18]. For example, resident surgery trainees when randomised to standard versus VR training before performing laparoscopic cholecystectomy, made fewer errors and were faster, requiring only half the time to reach the skill level of intermediately skilled surgeons [19–21]. A recent critical review of this issue concluded that skills during simulation can be successfully transferred to the OR. All of the studies reviewed focused on procedures that are less complicated than a RAH and were done using laparoscopic versus open surgery and none were conducted in sub-Saharan Africa.

One of the key recommendations put forth by the Lancet Oncology Commission on Global Cancer Surgery in 2015 was immediate onsite training of LMI general surgeons and general gynaecologists in a limited repertoire of cancer surgery procedures tailored to a country’s most common and surgically amenable cancers. In line with this recommendation, Parham and other cervical cancer experts in Africa developed a framework for identifying high-priority conditions and using core-competencies-based training of local health workers for targeted skills-building [22]. Using this framework, a novel surgical oncology educational curriculum was implemented in Zambia and Malawi to evaluate the time it would take to train local general gynaecologists to the level of surgical proficiency in performing RAH with bilateral pelvic lymphadenectomy, the most critical surgical procedure for the treatment of early cervical cancer. It was concluded that more intensive pre-training and more frequent mentoring following training could have led to surgical proficiency in less time, using fewer procedures and at significantly less cost. Web-based video telemonitoring systems use video camera recordings of live surgical procedures and computer vision in order to track physicians’ hand and surgical tool motion and analyse surgical dexterity [23].

Extrapolating from pilot study training of surgeons in Zambia and Malawi, it is estimated that it costs approximately $14,525 to train each surgeon, excluding salary, travel and other costs for the mentor. The time and financial investment required to train surgeons to proficiently perform radical total abdominal hysterectomies (TAHs) in LMI countries make it prohibitively expensive to scale-up these surgical procedures unless more effective and cost-efficient means are found to augment hands-on training. Virtual simulation may speed up the time required to acquire basic skills, and improvements in web-based video monitoring may help trained surgeons maintain newly acquired surgery skills. Building on our collective experience, we have sought to evaluate an innovative and cost-effective approach to improvement in skills for conducting radical surgery for the treatment of invasive cervical cancer by:

- comparison of a novel VR simulation-enhanced training approach to the traditional training approach,
- assessing the maintenance of trainee performance using a web-based video monitoring system and
- comparing the economic and financial costs of the virtual simulation-enhanced training and standard training for a surgeon to achieve proficiency in treating invasive cervical cancer.

## Technical development of low-cost VR surgery simulation

To develop a fully immersive VRSS for open approach to radical TAH, it was first necessary to develop the software and hardware in such a manner that this equipment could be affordably scaled and utilised in training centres in Zambia and beyond using off the shelf hardware. Here we describe the process and specifications required to build the surgical simulation for clinical studies and training.

### Sequencing the development

VR software for surgical simulations (VRSS) is required to have very high-quality visuals and believable surgeon interactions to be immersive and cost-effective to be scaled. The art assets need to be efficiently constructed in order to maintain technological specifications to run on the commercially available Oculus Rift Hardware at the desired frame rate and support the interactions required for the surgical procedure. The visuals of this VRSS approach to cancer surgery consist of everything seen by the user, including 3-D objects, modelled and designed for accuracy to ensure the proper training of surgical modules through VR using 1:1 scale matching of real-world elements. The development specifications also necessitated a high frame rate of not less than 60 fps per eye without sacrificing visual fidelity to ensure full immersion. The technical development team assigned three priorities for the project:

- Correct visual reproduction of organs, tools and hand positions would be achieved by thoroughly analysing real-world counter parts necessary for the training modules and the creation of all assets would use 1:1 scale so that the VR world scale matched their real-world scale counterparts.
- Object material properties would be physics-based and organic materials would be approximated based on physics-based engine technology to allow for immersion into a believable simulation and lighting would match that of the equipment specifications.
- Assets would be developed with the potential for modification or expansion into other types of surgical training, without negatively influencing development deadlines or affecting performance.

We also pre-set the hardware technical specifications of the final VRSS product to ensure its affordability, portability and robustness in resource-limited environments. The hardware consists of an Oculus Rift and a portable desktop with the recommended specifications: Graphics Card (NVIDIA GTX 1060/AMD Radeon RX 480 or greater); CPU (Intel i5-4590/AMD Ryzen 5 1500× or greater); Memory (8GB + RAM); Video Output (Compatible HDMI 1.3 video output) and USB Ports (3× USB 3.0 ports, plus 1× USB 2.0 port).

**Figure 1.**
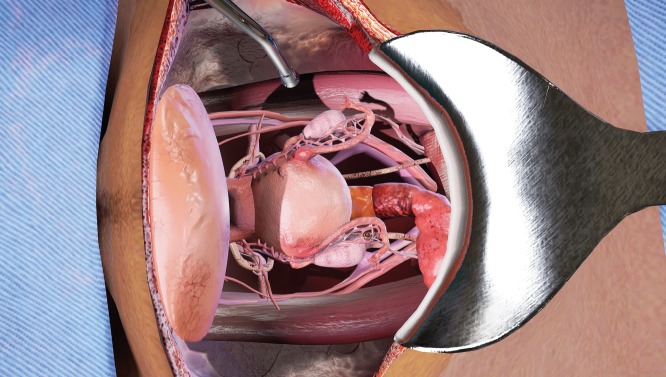
View of abdominal opening prepared for TAH procedure in the VR simulation.

**Figure 2.**
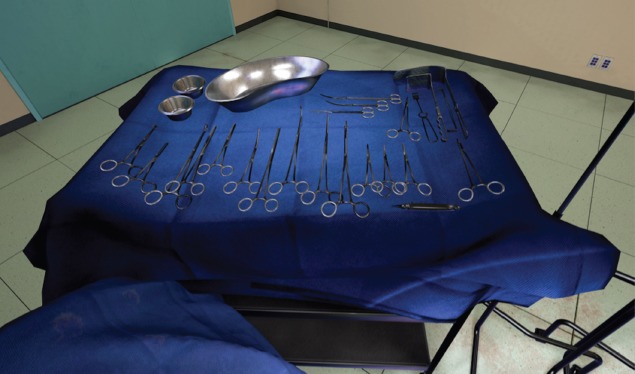
View of surgical instruments in the VR simulation.

The development phase was compartmentalised into three visual spaces based on their importance to the simulated surgery experience. The area of focus is the main surgical area of the patient. After the initial visual requirements were met, the priorities of completion for various aspects shifted to what is considered least likely to change. These are visuals that were sorted by revision pass order until completion. This also allowed development to continue without answers to questions, specific or general. As part of this development phase, a number of priorities were set for the development team:

- The opened surgical area of the patient, with the internal organs of the patient (Figure 1). A tray containing the surgical instruments required for the specific step in the surgical procedure (Figure 2). A tray to discard used instruments. The surgeon’s hands can move anywhere within this area.
- A wider area encapsulating the entire patient. The entire suite of tools and instruments. Operating theatre (OT) furniture that serves as boundaries for the surgeon. A monitor displaying simulated patient vitals and instructions. The surgeon’s hands can move anywhere within this area.
- The widest viewable area encapsulating the OT. Everything visible grounds the surgeon in our VR simulation by conveying true 1:1 scale and immersion through physics-based materials and lighting. The swivel lamps and overhead lamps. The surgeon’s hands cannot move anywhere within this area.

### Inside the VR world

**Figure 3.**
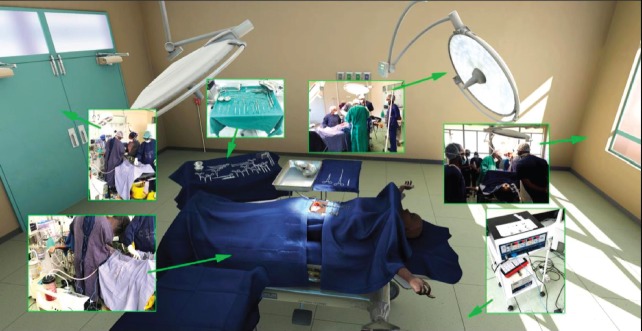
View of the virtual operating theater with callouts from real world references.

The location of the VR world is inside a virtual reconstruction of an OT found inside the University Teaching Hospital located in Lusaka, Zambia. The development team received photographic and video reference of the interior of the hospital, as well as the tools required for the specific training modules (Figure 3). This material was augmented through research of general OT and manufacturer specifications of medical products.

**Figure 4.**
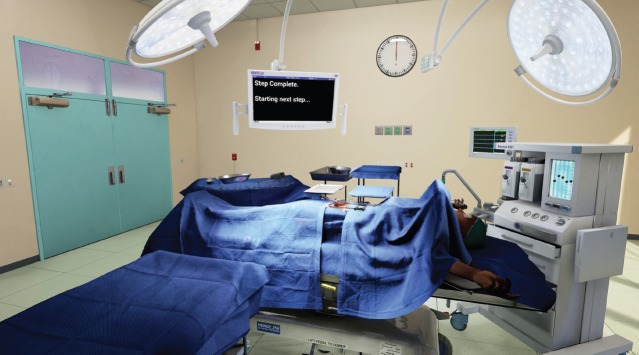
Wide view of virtual operating theater, shows user interface and potential for light position customization.

The OT is 7.6 m × 7.6 m room with a ceiling height of 3.4 m. These measurements fall between an averaged size for new and old world ORs. It was constructed modularly to allow for easy size adjustment during the early stages of the development. Procedural unwrapping and textures allowed it to quickly be de-modularised or sealed up seamlessly in order to lower its memory footprint while running (Figure 4). The artist conducted deep research into amalgamated equipment brand manufacturing specs for equipment and tools in order to complete the visuals and retain the proper scale for the surgical tools. A human hand was modelled to scale and mirrored to create the left and right hands of the surgeon. This hand was then rigged and skinned. This provided the potential to have a movement match for the surgeon’s real hand as the Oculus touch hardware progresses. These hand meshes represent blend shapes for multiple positions of the hands. The rigged hand model that generated each position eventually replaced the original models. The organs were constructed in 3-D following deep research into the human female anatomy. Although 3-D scans of human organs do exist, they are simply too inefficient to run in real time, especially at 120 fps. The organs were rigged with boxes representing connection points and structure to allow surgeons to manipulate, clamp, cut and suture the necessary ligaments, arteries and veins. The textures and materials of the anatomy are meant to simulate their real-world counterparts within the technical limitations of the project.

**Figure 5.**
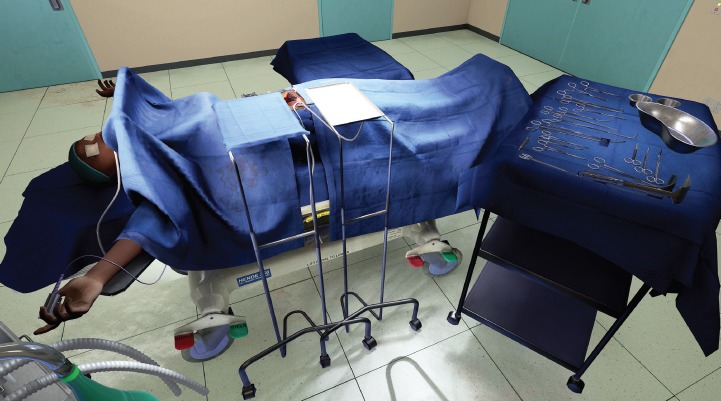
View of virtual operating theater from potential view of assisting surgeon or nurse.

All equipment and furniture were modelled after amalgamated manufacturer specifications of that type of product (Figure 5). The textures and materials were designed to match the reference images equally to the matched geometry. Additional specialised software was used to translate these elements from photos and construct them to match physically. Like everything else in this project, the user interface was designed to match real-world equivalents.

## Clinical development of VRSS for RAH

Most VRSS stops at the development point and is rapidly introduced into clinical practise with little rigorous testing. Few VR or AR approaches to surgery have been properly evaluated within the context of clinical studies or trials. In our approach to developing VRSS for RAH, the development-trial cycle was considered crucial to properly understand the impact and limitations of VRSS to augment surgical training. Here we describe our clinical study approach to post technical development of VRSS for RAH.

Surgical trainees were randomly assigned to one of two training conditions: a VR simulation-enhanced surgical training or traditional surgical (TS) training (Videos 1 and 2). This concluded with each surgical trainee being evaluated in the performance of a RAH under the guidance of an expert mentor surgeon blinded to their previous training. We aimed to develop a VR simulation training platform for the surgical treatment of invasive cervical cancer and compare the effectiveness of TS training (‘control condition’) to TS training-enhanced with VR simulation (‘intervention condition’) on the acquisition of the surgical technical skills needed to reach proficiency in treating invasive cervical cancer. This also included a bolt-on socio-economic evaluation of TS versus VR-enhanced training to reach surgical proficiency in treating invasive cervical cancer. In the ‘control’ condition (TS training), we modified the version of the cervical cancer surgery training curriculum developed and successfully used by the Gynaecologic Oncologists of Canada to intensively train a small number of locally identified gynaecologists to perform radical hysterectomy and pelvic lymphadenectomy that has been long used to train surgeons in LMI countries to perform RAH. The training consisted of online didactic lectures, readings and seven modules, with embedded videos that correspond to the surgical procedure. Trainees received a pre-test and post-test on the training materials and trainees continued reviewing the training over the course of 3 weeks and retaking tests until they received a passing proficiency score on the material. In the ‘intervention’ condition (VR simulation-enhanced surgical training), five critical steps in the RAH procedure were simulated to build surgical proficiency in executing the most difficult and complex aspects of the RAH, as determined by a consensus of senior gynaecologic oncology experts.

In the VRSS, the surgeon trainee stands at a table wearing the Oculus Rift headset and headphones, and an Oculus Touch hand controller in each hand. The Oculus Rift headset provides sensory input from a computer that runs the VR software allowing him or her to see the VR with an operating table, tray of surgical instruments and the patient with cervical cancer prepped and awaiting surgery. As in an actual surgical procedure, the virtual patient is covered, leaving exposed the section that will be the focus of the procedure. The surgeon can see their virtual arms and hands as he or she physically moves them on the virtual surgical field during the simulation and hears OR sounds as he or she advances through the operation. Also, as the surgeon moves the buttons of the hand controls, he or she can pick up the surgical instruments from the virtual surgical instrument tray and use them to complete specific actions in the RAH procedure on the virtual patient.

The surgeon is able to use both hands to perform related and unrelated actions, such as clamp in one hand and a scalpel in the other. Instructions are provided on a monitor above the operating table, and audio feedback is provided during the simulations, as appropriate, to guide the surgeon through the simulations. Each simulation lasts approximately 20 minutes and provides the surgeon with feedback on his or her accuracy on various portions of the procedure and an overall score on the simulation to indicate whether he or she has reached a level of proficiency as determined by the Senior Surgeon. Each VR pre-training session is recorded for review and reflection by the trainee. To compare the impact of TS versus VR conditions on surgical proficiency, trainees are assessed by the expert surgeon-mentors during both supervised training and direct hands-on training and using a reliable and valid structured method of rating surgical competency.

## Future evolution of AR/VR in building capacity and capability in cancer for LMICs

One of the crucial aspects of our work has been to build a high-fidelity VRSS that reflects the reality of the procedure and the ORs in Zambia (Video 1). For the latter, this has meant a faithful reproduction of the actual OR and instructions from a familiar and trusted mentor (Parham). We have also focused on VRSS of open procedures for surgery for cervical cancer. Indeed, open RAH has been shown to be superior in terms of outcomes compared to MIS in this setting [24, 25]. This suggests that VRSS for open procedures developed for low-middle income countries (LMICs) will also be important training tools in high-income settings where the dominance of MIS and robotic approaches is now coming under proper scrutiny, and in many cases found wanting [26]. The augmentation of surgical training using affordable VR and AR will have wide application in future training academies but needs to be carefully built and tested to ensure that it achieves the desired educational and learning outcomes. All too often new AR/VR technology is introduced and promoted without sufficient and rigorous pre-clinical and clinical research and testing. The opportunity exists now to build Centres of Excellence for Training Education in Cancer Surgery, as well as other procedures amenable to VR approaches that are deliverable in resource-limited settings and positively impact clinical skill to deliver better patient outcomes.

Commercial VR technology has also advanced significantly over the past few years and the industry is planning major new releases in 2019. This rapid development in VR technology is removing longstanding barriers to medical adoption. Importantly, head-mounted displays are becoming untethered and are light enough to be worn for extended periods of time, and processing power allows displays to keep up with human perception to prevent motion sickness [27]. Recent announcements by Oculus and HTC indicate that their new releases will support even lower cost commercial stand-alone VR headsets (down from $599 US with a PC to $399 US without the need for a PC) with higher resolution graphics, wireless display for interactions with six degrees of freedom and improved eye-tracking to quickly and precisely measure the direction a user is looking within a VR headset. In addition, inside-out tracking features remove the need for separate cameras or lasers around the VR interaction space. Together, these features broaden the applications for commercial VR technology in medical education and offer significant opportunities, especially for LMI countries.

## Conclusion

The call to arms issued by the WHO to eliminate cervical cancer cannot be achieved by vaccination alone. For decades to come, women diagnosed with cervical cancer will need access to safe, affordable and timely surgery, as well as other major modalities of cure and control such as radiotherapy. Increased screening for cervical cancer will also increase the diagnosis of benign disease, much of which will also require surgical intervention. The utilisation of new technologies such as VRSS as tools to help build wider capacity through in-country Centres of Excellence for Training and Education will play an increasingly important role. More widely, VR (and AR) have significant potential to help train workforce, augment safer surgery and ensure higher quality standards. But this can only be achieved if this technology is affordable and subject to rigorous research. This feasibility study to develop VRSS for RAH has shown that this is achievable, and the approach can be subjected to rigorous clinical studies. In order to further develop VR technology and understand the full impact on training outcomes, as well as integration into training programmes, the next steps will be to undertake a major multi-country clinical trial.

## Funding statement

This publication is funded through the UK Research and Innovation GCRF grant no: ES/P010962/1 and the Medical Research Grant GCRF_Zambia_UK MR/P025420/1 (comparison of traditional versus virtual simulation-enhanced training for scaling the cervical cancer surgery workforce in Zambia).

## Conflicts of interest

The authors declare they have no conflicts of interest.

## Figures and Tables

**Video 1.**
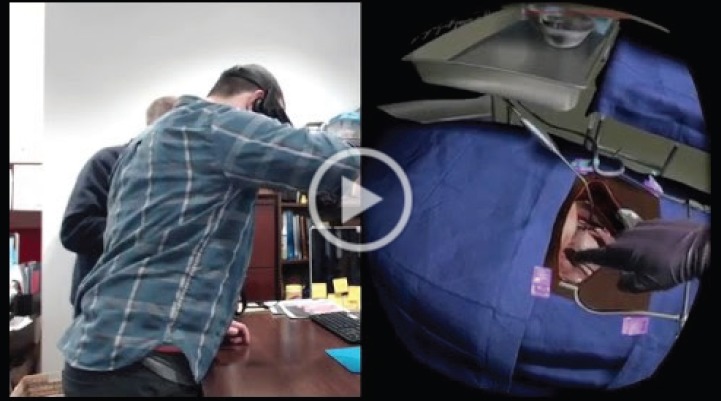
Virtual reality surgical simulation demonstration. To view this video, click here https://ecancer.org/journal/13/910-creating-a-low-cost-virtual-reality-surgical-simulation-to-increase-surgical-oncology-capacity-and-capability.php.

**Video 2.**
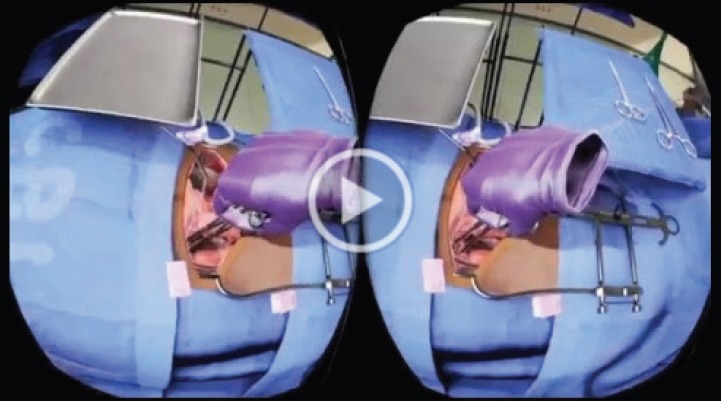
Deep Virtual Reality Training for TAH. To view this video, click here https://ecancer.org/journal/13/910-creating-a-low-cost-virtual-reality-surgical-simulation-to-increase-surgical-oncology-capacity-and-capability.php.
